# Laughter and Music

**Published:** 1859-05

**Authors:** 


### LAUGHTER AND MUSIC

Are alike in many points, both open the heart, wake up the affections, elevate our natures. Laughter ennobles, for it speaks forgiveness; music does the same, by the purifying influences which it exerts on the better feelings and sentiments of our being. Laughter banishes gloom; music—madness. It was the harp in the hands of the son of Jessee, which exorcised the evil spirit from royalty; and the heart that can laugh outright does not harbor treasons, stratagems and spoils.

Cultivate music then, put no restraint upon a joyous nature, let it grow and expand by what it feeds upon, and thus stamp the countenance with the sunshine of gladness, and the heart with the impress of a diviner nature, by feeding it on that "concord of sweet sounds" which prevails in the habitations of angels.

